# Immunotherapy with Intralesional Vitamin D Injection in Recalcitrant Wart at a Tertiary Care Center: A Descriptive Cross-sectional Study

**DOI:** 10.31729/jnma.8614

**Published:** 2024-06-30

**Authors:** Eliz Aryal, Jyoti Bhari, Prashanna Raj Shrestha

**Affiliations:** 1 Department of Dermatology, kathmandu Medical College, Sinamangal, Kathmandu, Nepal

**Keywords:** *immunotherapy*, *verruca*, *vitamin D*, *warts*

## Abstract

**Introduction:**

Recalcirant warts are resistant to conventional therapeutic option with high recurrence rate. In recent year, treatment of warts with different immunotherapeutic agent has shown good results, as it regulate epidermal cell proliferation and are involved in the formation of anti microbial peptides. Hence, this study was undertaken to evaluate the efficacy of immunotherapy with intralesional vitamin D in wart.

**Methods:**

A descriptive cross-sectional study was conducted from 1st January 2021 to 2nd February 2023 at Kathmandu Medical College after approval from the Institutional Review Committee (Reference number: 0110202002). Ninety - two patients with recalcitrant wart of varying sizes and duration were included in the study. Injection vitamin D ( 600000 IU, 15mg/ml) was injected about (0.2-0.5 ml) to the base of the wart. Maximum of five warts were injected per month, and was repeated after 4 weeks for 3 sessions.

**Results:**

Among 92 patient, complete response was seen in 70 patient (76.08%), partial response was seen in 17 patients ( 18.47%) and 5 patient (5.43%)showed no response. Mild pain as observed at the time of injection. Signs of hypervitaminosis D was not observed.

**Conclusions:**

Intralesional administration of Vitamin D is an effective treatment option for reclacitrant warts and is, highly effective, cost-efficient, with minimal adverse effects, and can be perfomed in our clinical set up.

## INTRODUCTION

Verruca vulgaris are skin disease caused by Human papilloma virus (HPV), is transmitted through direct contact with infected person or through contaminated materials. Virus initially target the basal layer and undergoes a latent phase of slow replication, as epidermis grows superficially, the virus induce hyperplasia and hyperkeratosis. Recalcitrant wart are resistant to conventional therapeutic option, despite treatment, continue to increase in size and number.^1^ Ablative modalities of therapy take months and many of them may result in pain, scarring, and recurrences. They only remove visible lesions; non-visible infected tissues are not targeted, resulting in a high chance of recurrence.^2^

In recent years, treatment of warts have included different immunotherapeutic agents like autogenous vaccine Candida albicans antigen, mumps, measles and rubella (MMR) vaccine, BCG vaccine.^3-5^ Intralesional Vitamin D3 injection was tried for the first time by Aktas et al with encouraging results.^6^ Vitamin D has property to regulate epidermal proliferation and cytokine production and serve as an immunotherapeutic agent with cost effective.^4^

This study was undertaken to evaluate the efficacy of immunotherapy with intralesional Vitamin D in recalcitrant warts.

## METHODS

This descriptive cross-sectional study was conducted from 1^st^ January 2021 to 2^nd^ February 2023 at kathmandu Medical College and Teaching Hospital after approval from the Institutional Review Committee (Reference number: 0110202002). All patients who attended the outpatient department for treatment of recalcitrant cutaneous wart diagnosed clinically by a registered dermatologist and meeting the inclusion criteria were enrolled in our study. The Inclusion criteria in our study include 1) Patients 12 to 70 years (The assend for (12-18) was taken, similarly a written consent was documented from all adult patient to enrolled in our study.) who gives consent 2) multiple warts, 3) Recalcitrant to conventional treatment.

Exclusion criteria consist of pregnancy, lactatingwomen, muco-cutaneous warts and immunosuppression. Baseline serum vitamin D and calcium level were done in all the patients and increased level of serum Vitamin D and calcium level were not included. Similarly 3 week wash out period was given for any conventional treatment used before.

Baseline evaluation was made at first visit and were recorded in structured questionnaire designed for this study, which include location, number, size and type of wart, duration of course, side effect of injection and clinical photographs. Using a 26 gauge insulin syringe 0.2.to 0.5 ml Vitamin D solution( 600,000 IU;15mg/ml) was slowly injected into base of each wart. According to number of warts, maximum 5 warts were injected in to base of each. The injection were performed at 4 weekly interval until compete resolution or for total 3 sessions.^1^ At each session clinical evaluation was done depending on number and size of wart. Response of Vit D injection was classified as^1^

1. Complete Response(CR)- Disappearance of wart and appearance of normal skin
2. Partial Response (PR) -50-99% Reduction in size and number
3. No Response (NR) - 0 - 40% Reduction in size.

Similarly, side effect was evaluated at each visit and was documented along with clinical photograph. An informed consent from each patients was sought prior to commencement of treatment. A total of 102 patients were recruited via census sampling method in two year duration.

Data was entered and analyzed using IBM SPSS Statistics software.

## RESULTS

Out of 102 patients, 10 patients were lost to follow up due to various reason and were not included in final statistical analysis. Amongst 92 patients 54(58.69%) were male and 38(41.30%) were female. The mean age of patients were 24.91 years (ranges,13-70 years).

In this study palmoplantar warts was observed in 33 (35.86%) patients follow by verruca vulgaris (Table 1). The duration of warts were from 6 months to 5 years, with mean duration being 16.5 months. The number of warts ranged from 3 to 30 in numbers. The dimension of warts range from 2 × 2 mm to 20 × 30 mm.

**Table 1:** Type of warts (n = 102).

Types	n (%)
Palmoplantar warts	33 (35.86)
Verruca vulgaris	30 (32.60)
Periungual warts	25 (27.17)
Plane warts	4 (4.34)

Complete Response (CR) was seenin 70 patients(76.08%) followed by Partial Response (PR) in 17(18.47%) and in 5 (5.43%) patientsNo Response(NR) was documented. According to type of wartspalmoplantar and verruca vulgarisshowed goodresponse with intralesional injection with Vitamin D3(Table 2).

**Table 2:** Treatment outcome for different type of warts.

Types	CR	PR	NR
Palmoplantar warts	28	5	0
Verruca vulgaris	20	6	4
Periungual warts	20	5	0
Plane warts	2	1	1

All patients had transient mild to moderate pain at the time of injection which lasted for half an hour. Edema and pain persist for 2-3 days in case of peri-ungual warts 25 (27.17%) and managed by NSAID. Similaly mild pain was also persist on patients with warts present over knuckle area of finger, and were advised to restrict the hand movement for 2-3 days.

No sign of hypervitaminosis D or systemic side effects were notice during regular follow up in each patients. Similarly cutaneous scarring and pigmentation were not observed. Out of 92 patient enrolled in our study only one patient developed cellulites at site of injection on third day of injection of Vitamin D3, and was managed by oral antibiotic for seven days.

## DISCUSSION

The result of this present study was matching with Akas and Manjunath by intralesional Vitamin D3 injection.^6,7^ Akas et al in 2014 used intra lesional Vitamin D3 for plantar warts. 7.5mg of Vitamin D3 injection was given monthly interval for 2 sessions in twenty patients. Complete clearance was reported in 80% at end of 8 weeks.^6^ Similarly in 2016 Study done in India, by manjunath fourty two patients in plamoplantar and common warts were enrolled and injection Vitamin D3 intra lesion was given interval between 2 week for maximum 4 session show complete clearance in 78.57 %. Both the study result were similar with our study. (Table 2) The response rate achieved in our study was 76% in maxmum of 3 session. There were no systemic complication with minimal local complication In this study, as we inject Vitamin D at the interval of 4 week for maximum of 3 session. This can be explained as treatment approach was similar and all above study were done in asian subcontinent with small sample size of patients and also study was not controlled.

Vitamin D, when use topically was found effective in treatment of warts.^8^ Successful treatment was documented in renal transplant recipient with use of topical Vitamin D3 in recalcitrant warts by Moscarelli et al.^9^ Similarly Egawa and Ono used Vitamin D3 topically in recalcitrant warts using half day occlusive dressing technique with good response.^10^ Effective clearance of warts was documented in all 17 patients following topical Vitamin D3 therapy.^11^

Various other antigen/vaccine are used as immunotherapy in verruca vulgaries. The response rate achieved in our study was (76.08%) in 3 session was superior to the result achieved with PPD (purified protein derivative),^12^ MMR(Measles mump rubella),^13^ and Bleomycin.^14^ Garg and Baveja reported that treatment with Mycobacterium W was superior to that of Vitamin D3, but the number of session was more, almost,^10^ as compare to their study which was 4. Furthermore, they reported local and systemic complication such as fever, redness, ulcer at site of injection.^15^

The study done is Turkey demonstrated normal level of vitamin D in serum with healthy control subjects comparing with verruca vulgaris patients, who had decreased in serum Vitamin D. In our study all subject enrolled had low Vitamin D level.^16^

Recalcitrant warts are frustrating and challenging to the patients and treating physician. The effect of Vitamin D in warts is still not completely understood, it act as controlling cell proliferation, differentiation via upregulation of vitamin D receptor of skin that leads to the activation of toll like receptor of macrophage and induction of antimicrobial peptides, lymphopiotin and cathelecidin. It also synthesis of IL Ia and IL6 resulting in decreased inflammation.^9^

Hence, Immunotherapy boosts the immune system to HPV virus leading to clearing of both treated and untreated warts.^9^

The limitations were single center study with using single treatment modalities and comperative, placebo treatment was not used.

## CONCLUSIONS

A excellent response was observe with Immunotherapy with Intralesional Vit D injection in Recalcitrant wart. Intralesional Vitamin D is a new upcoming therapeutic for recalcitrant warts. It is simple, well tolerable, effective, inexpensive method with negligible side effects, and can be performed in our clinical setup.
